# Case Report: Sustained complete response achieved in a patient with advanced HER-2 positive gastric cancer after 4 years of chemotherapy-free treatment

**DOI:** 10.3389/fonc.2026.1756461

**Published:** 2026-04-14

**Authors:** Xinyu Tong, Jieqiong Peng, Bo Liu

**Affiliations:** 1 Department of Oncology, Shandong First Medical University and Shandong Academy of Medical Sciences, Jinan, China; 2 Department of Oncology, Shandong Cancer Hospital and Institute, Shandong First Medical University and Shandong Academy of Medical Sciences, Jinan, China

**Keywords:** chemotherapy-free, complete response, HER2 bispecific antibody(BsAb), HER-2 positive, PD-L1/CTLA-4 bispecific antibody

## Abstract

**Background:**

Gastric cancer ranks fifth in both global incidence and mortality rates, with over half of the newly diagnosed cases worldwide occurring in China. Most patients are diagnosed at advanced stages, making surgical resection unfeasible. As a result, current treatment primarily relies on chemotherapy-based multimodal therapies. However, with ongoing research, chemotherapy-free treatment strategies are gaining increasing attention and have emerged as a promising avenue for the treatment of gastric cancer.

**Case presentation:**

A 42-year-old male was diagnosed with stage IV gastric adenocarcinoma and multiple liver metastases due to abdominal bloating. The patient was enrolled in a clinical study and received a combination of KN026 and KN046 targeted therapy with immunotherapy. After two years of treatment, a complete response was achieved.

**Conclusions:**

The combination of anti-HER-2 bispecific targeting therapy and PD-1/CTLA-4 bispecific immunotherapy demonstrated promising efficacy and a favorable safety profile in this patient with advanced HER2-positive gastric cancer, achieving durable complete response without chemotherapy. This case provides a rationale for further exploring chemotherapy-free strategies using dual bispecific antibodies in carefully selected patients.

## 1 Introduction

Gastric cancer ranks fifth globally in both cancer incidence and mortality rates, with over half of the newly diagnosed cases worldwide occurring in China (1). Many gastric cancer patients are already at an advanced stage at the time of diagnosis, losing the opportunity for surgical resection. As a result, the current treatment for gastric cancer is primarily focused on chemotherapy-based multidisciplinary approaches. TCGA research has identified four subtypes of gastric cancer: EBV-positive, microsatellite instability (MSI), genomically stable (GS), and chromosomal instability (CIN). Among these, EBV-positive and MSI gastric cancer patients can benefit from immunotherapy and generally have a better prognosis (2). In recent years, several new targets for targeted therapy in gastric cancer have been explored, such as FGFR, Claudin 18.2, c-MET, and NTRK. One such targeted therapy is zolbetuximab, a monoclonal antibody against Claudin 18.2. The SPOTLIGHT and GLOW trials have confirmed that zolbetuximab significantly improves progression-free survival (PFS) and overall survival (OS) in CLDN18.2-positive, HER2-negative, locally advanced unresectable, or metastatic gastric cancer patients (3, 4).

Globally, the HER2-positive rate in gastric cancer patients ranges from 7.3% to 20.2% (5). HER2 positivity is defined as immunohistochemistry (IHC) 3+ or IHC 2+ with fluorescence *in situ* hybridization (FISH) positive. For HER2-negative gastric cancer cases, chemotherapy combined with immunotherapy is the main treatment approach.The most well-established target for targeted therapy, HER2, is treated with the standard approach of combining the HER2-specific antibody trastuzumab with chemotherapy (6). Additionally, based on the results of the KEYNOTE-811 study, the combination of trastuzumab, chemotherapy, and pembrolizumab has become a first-line treatment for HER2-positive advanced gastric cancer patients with a CPS≥1.

In recent years, the new generation of antibody-drug conjugates (ADCs) has made significant advancements in the field of cancer treatment, owing to their precise targeting and exceptional bystander effects. Specifically, for HER2-positive advanced gastric cancer, ADC-based precision therapies have ushered in a new era of treatment. Trastuzumab deruxtecan (T-DXd) and RC48 have both been approved for the treatment of HER2-positive advanced gastric cancer as third-line or later therapies, and are also being explored in first-line treatment settings (7, 8). Immunotherapy, represented by immune checkpoint inhibitors (ICIs), has revolutionized the treatment landscape of various malignancies, offering long-term survival prospects for many patients. Studies have shown that programmed death-1 (PD-1) and its ligand, programmed death-ligand 1 (PD-L1), play a critical role in mediating tumor immune evasion. Tumor cells evade immune surveillance by upregulating PD-L1, which suppresses the activation, proliferation, and cytokine production of tumor-specific T cells, thereby facilitating tumor immune escape. In addition, cytotoxic T lymphocyte-associated antigen-4 (CTLA-4) inhibits T cell activation by trans-endocytosing the cluster of differentiation 80 (CD80) and CD86 molecules on the surface of antigen-presenting cells (APCs), thereby reducing their expression levels (9).

Recently, novel bispecific antibodies have emerged as promising therapeutic strategies. KN026 is a bispecific antibody that simultaneously binds to two distinct HER2 epitopes, potentially offering enhanced antitumor activity compared to traditional monoclonal antibodies. KN046 is a bispecific antibody targeting both PD-1 and CTLA-4, designed to synergistically reactivate T-cell-mediated immune responses. The combination of KN026 and KN046 represents a chemotherapy-free strategy that integrates dual HER2 blockade with dual immune checkpoint inhibition, providing a potential alternative to conventional chemotherapy in HER2-positive gastric cancer. Here, we report a case of advanced HER2-positive gastric cancer treated with this combination regimen.

## 2 Case report

In November 2021, a 42-year-old male presented to a local hospital with a six-month history of postprandial abdominal bloating without any obvious triggers, along with a reduced food intake. There were no symptoms of nausea or vomiting after eating. The patient reported radiating pain to the shoulder and back, but no abdominal pain. He did not experience fever, chills, headaches, or dizziness. A gastroscopy revealed a crater-like ulcer in the gastric antrum with overlying dirty coating and surrounding mucosal congestion and edema. A biopsy was taken, and the tissue was firm. The endoscopic diagnosis was gastric cancer, and pathology confirmed moderately differentiated adenocarcinoma in the gastric antrum.

Blood tests revealed an increase in CA19–9 to 82.9U/ml, CA72–4 to 15.2U/ml and CEA to 17.6ng/ml. Liver biochemistry shows elevated levels of ALP, LDH, and α-hydroxybutyrate dehydrogenase. Immunohistochemical analysis demonstrated positive HER2 expression with a score of 3+, pMMR status, and a PD-L1 CPS of 10.Contrast-enhanced computed tomography(CECT) revealed the gastric antrum wall shows irregular thickening, with an uneven mucosal surface and a blurred serosal surface. On contrast-enhanced imaging, there is heterogeneous enhancement. Enlarged lymph nodes are observed in the surrounding abdominal cavity, with the largest node measuring approximately 1.8 cm in short diameter. In the omental bursa, slightly enlarged lymph nodes are noted, with the largest measuring about 0.5 cm in short diameter. Multiple low-density nodules and masses are seen in the liver, with indistinct borders. The largest mass measures approximately 7.1cm x 5.1cm and shows ring-like enhancement on contrast-enhanced imaging.

The patient was diagnosed with cT4NxM1 stage IV gastric adenocarcinoma with abdominal lymph node metastasis and multiple liver metastases. Based on the opinions of the multidisciplinary team consultation, it is recommended that the patient undergo chemotherapy or chemotherapy in combination with targeted therapy. Given the compelling efficacy data of KN026 in combination with KN046 in HER2-positive gastric cancer and the patient’s refusal of chemotherapy due to fear of chemotherapy-related adverse effects, a chemotherapy-free regimen was selected.The patient, after screening, met the criteria for the “Phase II Clinical Study on the Efficacy, Safety, and Tolerability of KN026 Combined with KN046 for HER-2 Positive Solid Tumors (Protocol No: KN026-203).” Contraindications were excluded, and from December 14, 2021, the patient was administered KN026 combined with KN046 for immune + targeted therapy.

**Figure 1:**
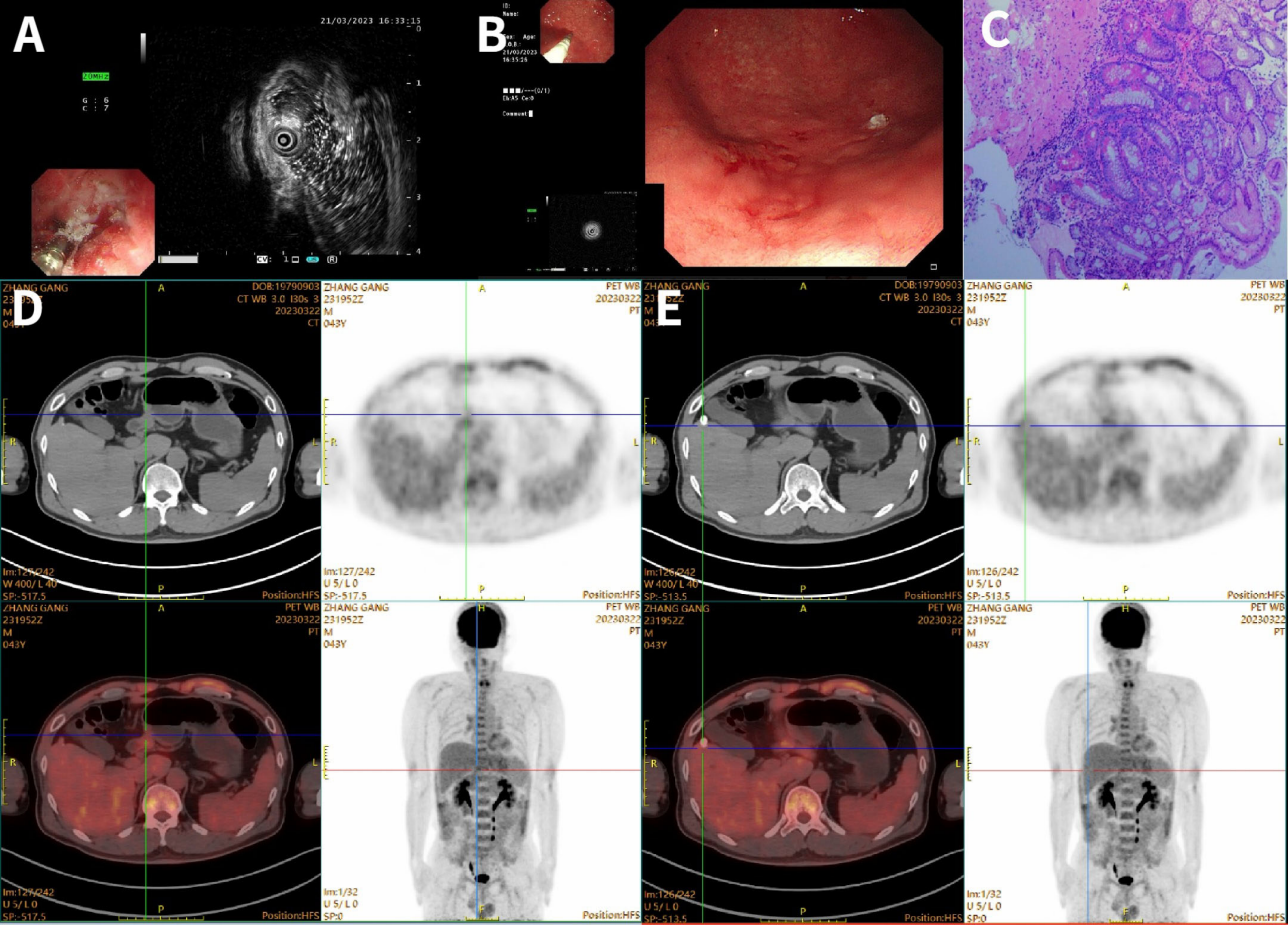
**(A, B)** Endoscopic ultrasound shows involvement of the muscularis propria; rough gastric antral mucosa, pathology pending; chronic atrophic gastritis. **(C)** Hematoxylin and eosin (H&E) staining: (gastric antrum greater curvature biopsy) chronic atrophic gastritis with intestinal metaplasia of the glands. **(D, E)** PET/CT images of the primary gastric cancer lesion and liver metastases.

**Figure 2:**
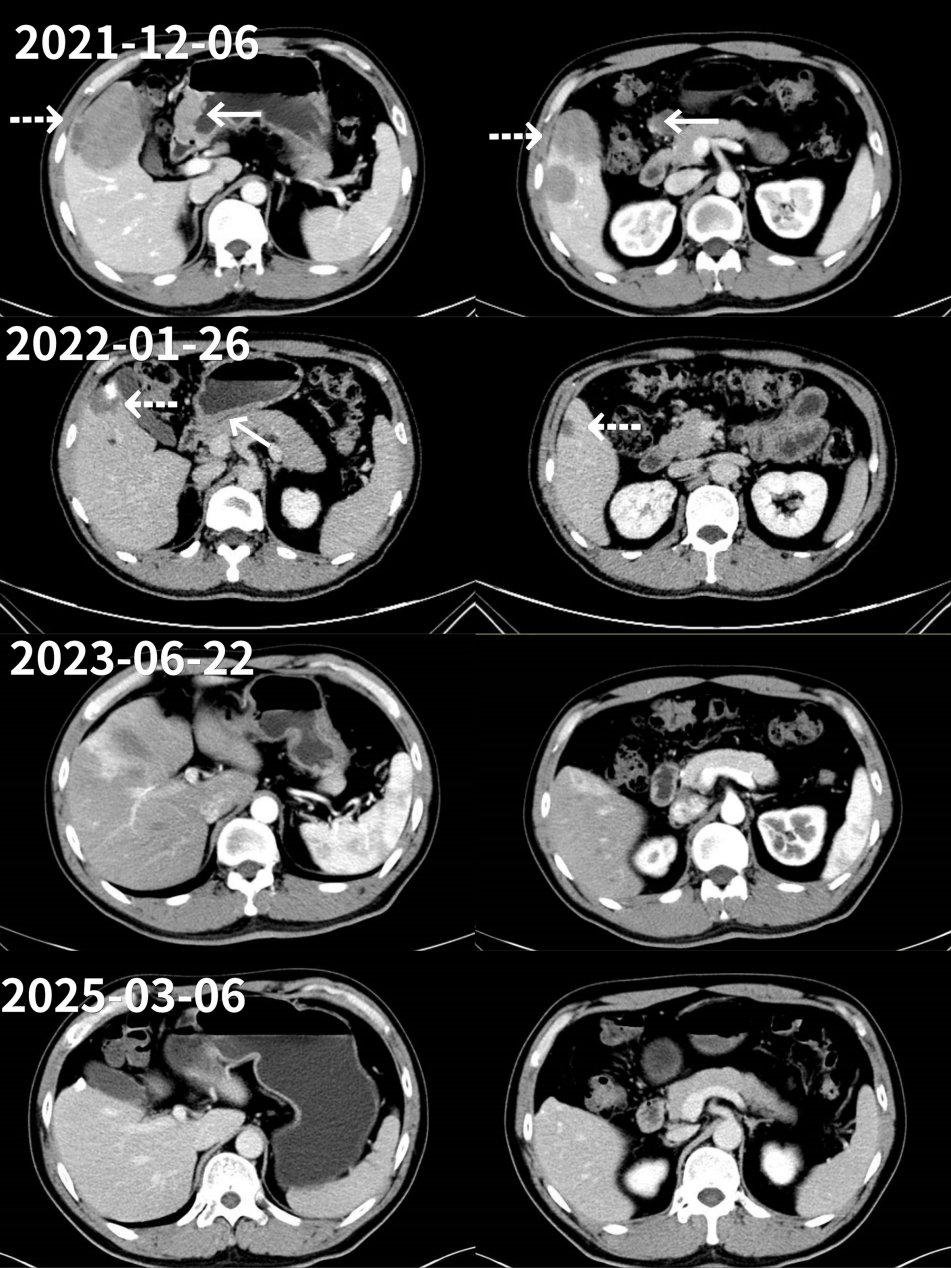
CT images obtained on December 6, 2021, upon the patient’s admission, showing multiple hypodense nodules and masses in the liver with ill-defined borders. The largest mass measures approximately 7.1cm×5.1cm, and shows rim enhancement on contrast-enhanced scans.The remaining images are follow-up CT scans during the treatment period. Comparison shows that the patient’s liver metastatic lesions gradually improved and eventually disappeared completely.

The achievement of complete response (CR) was confirmed by PET/CT and histopathological examination. Following 23 cycles of KN026 plus KN046 treatment, an endoscopic ultrasonography performed on March 21, 2023, revealed the following: clinical post-treatment status for gastric cancer, with involvement of the muscularis propria observed; rough mucosal surface in the gastric antrum, requiring pathological confirmation; and chronic atrophic gastritis. Pathological analysis of the gastric biopsy (greater curvature of the antrum) indicated chronic atrophic gastritis with intestinal metaplasia.A whole-body PET/CT scan on March 22, 2023, indicated no abnormal metabolic activity in previously documented gastric cancer with peritoneal lymph node and hepatic metastases, consistent with metabolic complete remission following treatment (**Figure 1**). Based on the endoscopic and PET/CT findings, the patient was assessed as having achieved CR. After two cycles of treatment, the patient’s follow-up CT showed significant improvement of the lesion. Subsequent imaging evaluations indicated continued stability after the initial improvement. A follow-up CT scan on March 6, 2025, confirmed: 1. No definite evidence of primary gastric lesion compared to the previous examination (November 7, 2024); 2. No visible abdominal lymph node or hepatic metastases. Overall treatment efficacy was evaluated as CR (**Figures 1**, **2**).

After 21 cycles of combined treatment with KN026 and KN046, the patient achieved complete response. The patient declined surgery and is continuing medication treatment.

## 3 Discussion

Currently, chemotherapy remains the cornerstone of gastric cancer treatment. Compared to best supportive care, it plays a role in symptom relief, prolonging survival, and improving quality of life (10). For a long time, the first-line treatment for unresectable advanced or recurrent gastric cancer, or gastroesophageal junction cancer, has been fluorouracil and platinum-based chemotherapy, a standard that has changed little over the past decade.

The commonly used chemotherapy regimens for advanced gastric cancer are mainly two-drug combinations of fluoropyrimidine and platinum-based agents. Common regimens include XELOX (oxaliplatin + capecitabine), FOLFOX (oxaliplatin + leucovorin + fluorouracil), and PF (cisplatin + fluorouracil), among others (11, 12). However, although these chemotherapy drugs have shown some effectiveness in inhibiting and killing tumor cells, their toxic effects on normal tissues and organs cannot be ignored. This toxicity often becomes a major barrier, limiting drug dosage and impacting treatment efficacy.

In light of these challenges, the development of active and well-tolerated regimens may offer an attractive alternative for patients with advanced gastric cancer. Targeted therapies, including anti-HER2 agents, anti-angiogenic drugs, and other biomarker-directed treatments, have demonstrated promising efficacy in treating gastric cancer, with particularly notable outcomes observed in patients with high biomarker expression. In recent years, immunotherapeutic approaches such as immune checkpoint inhibitors (e.g., PD-1/PD-L1 inhibitors) have achieved remarkable success across multiple cancer types, marking the advent of the immunotherapy era in oncology. With the advancement of targeted therapy, the ToGA study showed that the combination of trastuzumab with chemotherapy extended median overall survival from 11.8 to 16.0 months. Subsequent trials such as CheckMate 649 have further established immunotherapy as a viable option in the first-line setting. These novel therapeutic strategies, which combine chemotherapy with targeted and/or immune-based agents, have significantly improved both progression-free survival (PFS) and overall survival (OS), yielding impressive clinical outcomes (**Table 1**).

**Table 1:** First-line treatment for advanced HER2-positive gastric cancer.

Trial name/NCT number	Phase	Interventions	Participants(n)	ORR (%)	mPFS (month)	mOS (month)
ToGA (6)	III	Trastuzumab +Chemotherapy(Capecitabine/Fluorouracil+Cisplatin)	HER2-positive AG/GEJC(n=594)	47.0%	6.7	13.8
		Capecitabine/Fluorouracil+Cisplatin		35.0%	5.5	11.1
NCT02954536 (13)	II	Pembrolizumab + Trastuzumab+Oxaliplatin or Cisplatin+Capecitabine or 5-FU	HER2-positive metastatic GEC(n=37)	91%	13	27.3
PANTHERA (14)	Ib/II	Pembrolizumab +Trastuzumab+Capecitabine+Cisplatin	HER2-positive AG/GEJC(n=43)	76.7%	8.6	19.3
KEYNOTE-811 (15)	III	Pembrolizumab + Trastuzumab + Chemotherapy(5-FU+Cisplatin or Capecitabine+Oxaliplatin)	HER2-positive AG/GEJA(n=698)	72.6%	10	20
		placebo +Trastuzumab + Chemotherapy(5-FU+Cisplatin or Capecitabine+Oxaliplatin)		60.1%	8.1	16.8
Ni-HIGH (16)	Ib	nivolumab + trastuzumab + S-1/capecitabine+ oxaliplatin	HER2−positive AGC (n=42)	76.20%	-	-
JACOB (17)	III	Pertuzumab + Trastuzumab+chemotherapy(Cisplatin + Capecitabine/5-FU)	HER2-positive GC/GEJC (n=780)	56.7%	8.5	17.5
		placebo+Trastuzumab + chemotherapy(Cisplatin + Capecitabine/5-FU)		48.3%	7	14.2
TRIO-013/LOGiC (18)	III	lapatinib+CapeOx	HER2-amplified AGEJA (n=545)	53%	6	12.2
		placebo+CapeOx		39%	5.4	10.5
DESTINY-Gastric03 (7)	Ib/II	T-DXd	HER2-expressing advanced/metastatic G/GEJ/E AC (n=307)	48.8%	9	18
		T-DXd+Pembrolizumab		63.4%	8	16
		T-DXd+5-FU/cape +Pembrolizumab		58.1%	10	23
		T-DXd+5-FU/cape		78.0%	20	23
		T-DXd+5-FU/cape+Pembrolizumab(low dose)		12.5%	NA	NA
		Trastuzumab+5-FU/cape+cisplatin/oxaliplatin		75.9%	12	18
ARTEMIDE-Gastric01 (19)	III	Rilvegostomig + T-DXd +capecitabine or 5-fluorouracil (5-FU)	HER2-positive PD-L1 CPS ≥1 advanced/metastatic GC/GEJC (n≈840)	Ongoing	Ongoing	Ongoing
		pembrolizumab + trastuzumab + 5-FU and cisplatin (FP) or capecitabine and oxaliplatin (CAPOX)		Ongoing	Ongoing	Ongoing
		Rilvegostomig+trastuzumab+FP or CAPOX		Ongoing	Ongoing	Ongoing
RCTS (8)	II	RC48 + tislelizumab + S-1	HER2-overexpressing metastatic/unresectable GC/GEJC (n=47)	95.0%	not reached(9-month PFS rates were 80.8%)	not reached(9-month OS rates were 83.8%)
NCT06109467 (20)	II	mFOLFOX+trastuzumab + pembrolizumab+neratinib	HER2-positive stage IV GEC (n=30)	onging	Ongoing	Ongoing
AIO INTEGA (21)	II	trastuzumab+nivolumab+mFOLFOX6	HER2-positive metastatic EGA (n=97)	56%	10.7	21.8
		trastuzumab+nivolumab+ipilimumab		32%	3.2	16.4
MAHOGANY (22)	II/III	Margetuximab+Retifanlimab	HER2+/PD-L1+ unresectable/metastatic GEJA (n=43)	53%	11.4	not reached
NCT04276493 (23)	Ib/II	Zanidatamab + tislelizumab +CAPOX	HER2+ unresectable advanced/metastatic GC/GEJ (n=33)	75.8%	16.7	-
NCT03929666 (24)	II	zanidatamab + mFOLFOX6 or CAPOX or FP	HER2-expressing advanced/metastatic GEA (n=46)	76.2%	12.5	36.5
HERIZON-GEA-01 (25)	III	trastuzumab+chemotherapy(CAPOX or FP)	HER2-positive advanced/metastatic G/GEJ/E AC (n=714)	Ongoing	Ongoing	Ongoing
		zanidatamab + chemotherapy ± tislelizumab		Ongoing	Ongoing	Ongoing
KN026-203 (26)	II	KN026+ KN046	HER2-positive advanced/unresectable/metastatic GC/GEJ (n=31)	77.8%	-	-
(27)	Ib/II	KN026+ KN046	HER2-positive non-breast cancer (n=113)	55.6%(In the 1L HER2-positive GC subgroup:78.9%)	In the 1L HER2-positive GC subgroup:11	not reached
(28)	II	inetetamab + SOX	HER2-positive AGC/GEJ AC (n=38)	50%	8.5	15.3
		trastuzumab+ SOX		42%	7.3	14.3

A study led by Professor Shen Lin’s team evaluated the efficacy and safety of the bispecific anti-HER2 antibody KN026 in combination with the anti-CTLA-4/PD-L1 bispecific antibody KN046 in patients with advanced HER2-positive solid tumors (26, 29). Preliminary efficacy and safety data previously released indicated that this regimen exhibits potential clinical benefits surpassing existing standard therapies, along with initial yet profound antitumor activity. Results from the Phase Ib and Phase II clinical trials have recently been announced. Among the 108 patients evaluable for efficacy, the overall objective response rate (ORR) was 55.6%.In the HER2-positive gastric cancer (GC) subgroup, 38 patients received the combination as first-line treatment, of whom 30 achieved an objective response, resulting in an ORR of 78.9% and a disease control rate (DCR) of 89.5%. Additionally, 27 HER2-positive GC patients received KN026 plus KN046 as second-line or later therapy. In this group, 12 patients achieved a partial response (PR) and 11 had stable disease (SD), yielding an ORR of 44.4% and a DCR of 85.2%. The median duration of response (DoR) was 10.8 months, and the median progression-free survival (PFS) was 5.3 months.In another subgroup of patients with other HER2-positive solid tumors (34 patients), the ORR was 52.9% (27). These data further validate the promising efficacy and favorable safety profile of KN026 combined with KN046 in HER2-positive cancers.These accumulating data support the feasibility and growing adoption of chemotherapy-free regimens in selected patients with HER2-positive advanced gastric cancer.

However, this patient achieved remarkable therapeutic outcomes with only combined immunotherapy and targeted treatment, demonstrating effective control of the primary lesion and complete resolution of metastatic sites. These findings provide new insights into the treatment of HER2-positive advanced gastric cancer. Compared with conventional chemotherapy, chemotherapy-free regimens offer more precise targeting and fewer adverse effects, thereby overcoming the limitations associated with traditional chemotherapy. Multiple studies have suggested that anti-HER2 therapy and immune checkpoint inhibitors have synergistic effects.

The patient was treated with a combination therapy consisting of KN026 (an anti-HER2 bispecific antibody) and KN046 (a PD-L1/CTLA-4 bispecific antibody). KN026 is a HER2 bispecific antibody generated using a heterodimeric Fc platform, capable of simultaneously binding two non-overlapping epitopes of HER2 (ECD2 and ECD4). This dual binding leads to potent blockade of HER2 signaling, demonstrating superior efficacy compared to the combination of trastuzumab and pertuzumab, along with higher binding affinity. As a result, KN026 exhibits enhanced antitumor activity in HER2-positive tumor cell lines. Moreover, KN026 also shows inhibitory effects in tumors with low to moderate HER2 expression and in trastuzumab-resistant cell lines (30). In a study by Xu et al. (31), 45 patients with locally advanced or metastatic HER2-expressing gastric or gastroesophageal junction cancer were categorized into HER2-high, HER2-low, and HER2-negative groups. The HER2-high group achieved an objective response rate (ORR) of 56% and a median duration of response of 9.7 months, while the HER2-low group had an ORR of 14%. Both median overall survival (mOS) and ORR were significantly prolonged in the HER2-high group (mOS: 16.3 months vs. 9.6 months; ORR: 56% vs. 14%). The incidence of serious adverse events was 20%. These results indicate that KN026 has promising antitumor efficacy and a favorable safety profile in patients with advanced HER2-positive gastric or gastroesophageal junction cancer. KN026 was well-tolerated, with the majority of toxicities being low-grade and transient. Significant antitumor activity was also observed in HER2-positive patients who had progressed after prior trastuzumab-based therapy. Therefore, KN026 has the potential to serve as a chemotherapy-free option and holds great promise for combination therapy development in patients with advanced HER2-positive gastric or gastroesophageal junction cancer who have received at least one prior line of standard treatment (**Table 2**).

**Table 2:** Second-line and subsequent-line treatment for advanced HER2-positive gastric cancer.

Trial name/NCT number	Phase	Interventions	Participants(n)	ORR (%)	mPFS (month)	mOS (month)
GATSBY (32)	II/III	trastuzumab emtansine	HER2-positive AG/GEJA (n=70)	20.6%	2.7	7.9
		taxane(paclitaxel/docetaxel)		19.6%	2.9	8.6
TyTAN (33)	III	Lapatinib + paclitaxel	HER2-amplified GC (n=261)	27.0%	5.4	11
		paclitaxel		9.0%	4.4	8.9
NCT01522768 (34)	II	afatinib+Paclitaxel	HER2+ EGA (n=42)	9.5%	-	-
HER-RAM (35)	Ib/II	Trastuzumab + Ramucirumab + Paclitaxel	HER2-positive AG/GEJC(n=50)	54%	7.1	13.6
T-ACT (36)	II	paclitaxel+ trastuzumab	HER2-positive AG/GEJC (n=91)	33.0%	3.7	10
		paclitaxel		32.0%	3.2	10
CP-MGAH22–05 (37)	Ib/II	Margetuximab + pembrolizumab	HER2-positive unresectable locally advanced or metastatic GEA (n=95)	18.5%	2.73	12.48
NCT03925974 (38)	II	HER2 high-level cohort (Cohort 1: IHC3+ or IHC 2+ ISH+) : KN026	AGC/GEJC (n=45)	56.0%	8.3	16.3
		HER2 low-level cohort (Cohort 2: IHC 1+/2+ ISH- or IHC 0/1+ISH+): KN026		14.0%	1.4	9.6
NCT05427383 (39)	II	KN026+paclitaxel/irinotecan	HER2-positive GC/GEJC (n=39)	40.0%	8.6	13.2
KC-WISE (40)	II/III	KN026 + Paclitaxel/Docetaxel/Irinotecan	HER2-positive advanced unresectable or metastatic GC/GEJ AC(n=188)	55.8%	7.1	19.6
		placebo+Paclitaxel/Docetaxel/Irinotecan		10.8%	2.7	11.5
NCT05190445 (41)	II	PRS-343+ramucirumab + paclitaxel or PRS-343+tucatinib	G/GEJA (n=80)	ongoing		
DESTINY-Gastric01 (42)	II	Trastuzumab deruxtecan(DS-8201)	HER2-positive G/GEJA(n=187)	51.0%	5.6	12.5
		irinotecan/paclitaxel		14.0%	3.5	8.4
DESTINY-Gastric02 (43)	II	T-DXd	HER2+ unresectable/metastatic G/GEJC(n=79)	41.8%	5.6	12.1
DESTINY-Gastric04 (44)	III	T-DXd	HER2+ unresectable/metastatic GC/GEJA(n=494)	44.3%	6.7	14.7
		ramucirumab+paclitaxel		29.1%	5.6	11.4
DESTINY-Gastric06 (45)	II	T-DXd	HER2+ AG/GEJA(n=95)	29.0%	6	11
RC48-C013 (46)	I	RC48+toripalimab	AG/GEJC(n=30)or other solid tumours(n=26)with HER2 IHC≥1 or ISH positivity	43.0%	6.2	16.8
RC48-C008 (47)	II	RC48	HER2-overexpressing, locally advanced or metastatic G/GEJC(n=125)	24.8%	4.1	7.9
ACE-Gastric-01 (48)	I	ARX788	HER2-positive advanced G/GEJA(n=30)	37.9%	4.1	10.7
MOUNTAINEER-02 (49)	II/III	tucatinib/placebo ± trastuzumab+ramucirumab+paclitaxel	locally-advanced unresectable or metastatic HER2+ GEC(n=17)	37.5%/100%	4.1/12.2	-
ASPEN-06 (50)	II/III	evorpacept (ALX148)+trastuzumab + ramucirumab + paclitaxel	HER2+ advanced or metastatic GC(n=127)	40.3%	-	-
		trastuzumab + ramucirumab + paclitaxel		26.6%	-	-
NCT04446260 (51)	I	SHR-A1811	HER2-expressing or mutated unresectable, advanced, or metastatic solid tumors(n=307)	59.9%	-	-

KN046 is a novel recombinant humanized bispecific antibody-fusion protein targeting PD-L1 and CTLA-4. It concurrently blocks both PD-1 and CTLA-4 pathways, achieving dual immune checkpoint blockade, which enhances T cell-mediated antitumor immunity and promotes the immune system’s attack on cancer cells (52). Clinical studies of KN046 in non-small cell lung cancer and triple-negative breast cancer have shown promising progress, and trials in gastric cancer are currently ongoing, with further results awaited. Margetuximab is an Fc-optimized monoclonal antibody targeting HER2 with enhanced antibody-dependent cell-mediated cytotoxicity (ADCC) function. The combination of margetuximab and pembrolizumab achieved an objective response rate (ORR) of 35.7% in patients with HER2 IHC3+ gastric/gastroesophageal junction cancer (GC/GEJC), and the ORR reached 63.6% in the PD-L1+/HER2 IHC3+ subgroup, suggesting a synergistic effect between anti-HER2 therapy and immune checkpoint blockade (53).

This case of durable complete response contributes to the accumulating evidence favoring chemotherapy-free approaches for carefully selected patients with HER2-positive advanced gastric cancer. Such strategies are especially valuable for individuals who are ineligible for or decline conventional chemotherapy, have contraindications to cytotoxic agents, or prioritize quality of life. Meanwhile, several bispecific antibody-based regimens are progressing through clinical development(**Tables 1**, **2**). These developments collectively indicate a broader paradigm shift toward incorporating bispecific antibodies into frontline treatment strategies.

## 4 Conclusion

In summary, a patient with advanced HER2-positive gastric adenocarcinoma achieved complete response following treatment with a combination of an anti-HER2 bispecific antibody and a PD-1/CTLA-4 bispecific immune checkpoint inhibitor. This chemotherapy-free regimen offers more precise targeting and a reduced incidence of adverse effects, thereby overcoming the limitations associated with conventional chemotherapy. The present case illustrates the clinical benefits of this approach and suggests its potential as a first-line treatment option for HER2-positive patients, though further clinical trials are required to confirm this finding.

## Data Availability

The original contributions presented in the study are included in the article/supplementary material. Further inquiries can be directed to the corresponding authors.

## References

[1] Bray F Laversanne M Sung H Ferlay J Siegel RL Soerjomataram I . Global cancer statistics 2022: GLOBOCAN estimates of incidence and mortality worldwide for 36 cancers in 185 countries. CA: A Cancer J For Clin. (2024) 74:229–63. doi: 10.3322/caac.21834. PMID: 38572751

[2] Network CGAR . Comprehensive molecular characterization of gastric adenocarcinoma. Nature. (2014) 513:202. doi: 10.1038/nature13480. PMID: 25079317 PMC4170219

[3] Shitara K Lordick F Bang Y-J Enzinger P Ilson D Shah MA . Zolbetuximab plus mFOLFOX6 in patients with CLDN18.2-positive, HER2-negative, untreated, locally advanced unresectable or metastatic gastric or gastro-oesophageal junction adenocarcinoma (SPOTLIGHT): a multicentre, randomised, double-blind, phase 3 trial. Lancet. (2023) 401:1655–68. doi: 10.1016/s0140-6736(23)00620-7. PMID: 37068504

[4] Shah MA Shitara K Ajani JA Bang Y-J Enzinger P Ilson D . Zolbetuximab plus CAPOX in CLDN18.2-positive gastric or gastroesophageal junction adenocarcinoma: the randomized, phase 3 GLOW trial. Nat Med. (2023) 29:2133–41. doi: 10.1038/s41591-023-02465-7. PMID: 37524953 PMC10427418

[5] Abrahao-MaChado LF Scapulatempo-Neto C . HER2 testing in gastric cancer: An update. World J Gastroenterol. (2016) 22:4619. doi: 10.3748/wjg.v22.i19.4619. PMID: 27217694 PMC4870069

[6] Bang Y-J Van Cutsem E Feyereislova A Chung HC Shen L Sawaki A . Trastuzumab in combination with chemotherapy versus chemotherapy alone for treatment of HER2-positive advanced gastric or gastro-oesophageal junction cancer (ToGA): a phase 3, open-label, randomised controlled trial. Lancet. (2010) 376:687–97. doi: 10.1016/s0140-6736(10)61121-x. PMID: 20728210

[7] Janjigian YY Viglianti N Liu F Mendoza-Naranjo A Croydon L . A phase Ib/II, multicenter, open-label, dose-escalation, anddose-expansion study evaluating trastuzumab deruxtecan (T-DXd, DS-8201) monotherapy and combinationsin patients with HER2-overexpressing gastric cancer (DESTINY-Gastric03). AmSoc Clin Oncol. (2021). 39(3_suppl):TPS261. doi: 10.1200/JCO.2021.39.3_suppl.TPS261

[8] Li S Liu Z Liu Y Li K Cong L Cao F . Efficacy of disitamab vedotin (RC48) plus tislelizumab and S-1 asfirst-line therapy for HER2-overexpressing advanced stomach or gastroesophageal junctionadenocarcinoma: A multicenter, single-arm, phase II trial (RCTS). Am SocClin Oncol. (2024). 42(16_suppl):4009. doi: 10.1200/JCO.2024.42.16_suppl.4009

[9] Zhang H Dai Z Wu W Wang Z Zhang N Zhang L . Regulatory mechanisms of immune checkpoints PD-L1 and CTLA-4 in cancer. J Exp Clin Cancer Res. (2021) 40:184. doi: 10.1186/s13046-021-01987-7. PMID: 34088360 PMC8178863

[10] Wagner AD Grothe W Haerting J Kleber G Grothey A Fleig WE . Chemotherapy in advanced gastric cancer: a systematic review and meta-analysis based on aggregate data. J Clin Oncol. (2006) 24:2903–9. doi: 10.1200/jco.2005.05.0245. PMID: 16782930

[11] Ajani JA D’Amico TA Bentrem DJ Chao J Cooke D Corvera C . Gastric cancer, version 2.2022, NCCN clinical practice guidelines in oncology. J Natl Compr Cancer Netw. (2022) 20:167–92. doi: 10.6004/jnccn.2022.0008. PMID: 35130500

[12] Wang FH Zhang XT Li YF Tang L Qu XJ Ying JE . The Chinese Society of Clinical Oncology (CSCO): clinical guidelines for the diagnosis and treatment of gastric cancer, 2021. Cancer Commun. (2021) 41:747–95. doi: 10.1002/cac2.12193. PMID: 34197702 PMC8360643

[13] Janjigian YY Maron SB Chatila WK Millang B Chavan SS Alterman C . First-line pembrolizumab and trastuzumab in HER2-positive oesophageal, gastric, or gastro-oesophageal junction cancer: an open-label, single-arm, phase 2 trial. Lancet Oncol. (2020) 21:821–31. doi: 10.1016/s1470-2045(20)30169-8. PMID: 32437664 PMC8229851

[14] Rha SY Lee C Kim HS Kang B Jung M Kwon WS . A multi-institutional phase Ib/II trial of first-line tripletregimen (Pembrolizumab, Trastuzumab, Chemotherapy) for HER2-positive advanced gastric andgastroesophageal junction cancer (PANTHERA Trial): Molecular profiling and clinicalupdate. Am Soc Clin Oncol. (2021).39(3_suppl):218. doi: 10.1200/JCO.2021.39.3_suppl.218

[15] Janjigian YY Kawazoe A Bai Y Xu J Lonardi S Metges JP . Pembrolizumab plus trastuzumab and chemotherapy for HER2-positive gastric or gastro-oesophageal junction adenocarcinoma: interim analyses from the phase 3 KEYNOTE-811 randomised placebo-controlled trial. Lancet. (2023) 402:2197–208. doi: 10.1016/s0140-6736(23)02033-0. PMID: 37871604

[16] Takahari D Shoji H Minashi K Hara H Chin K Oki A . Safety and early efficacy results of a phase Ib study of nivolumabplus trastuzumab with S-1/capecitabine plus oxaliplatin for HER2-positive advanced gastric cancer(Ni-HIGH study). Am Soc Clin Oncol. (2022)40(4_suppl):276. doi: 10.1200/JCO.2022.40.4_suppl.276

[17] Tabernero J Hoff PM Shen L Ohtsu A Shah MA Siddiqui A . Pertuzumab, trastuzumab, and chemotherapy in HER2-positive gastric/gastroesophageal junction cancer: end-of-study analysis of the JACOB phase III randomized clinical trial. Gastric Cancer. (2023) 26:123–31. doi: 10.1007/s10120-022-01335-4. PMID: 36066725 PMC9813086

[18] Hecht JR Bang Y-J Qin SK Chung HC Xu JM Park JO . Lapatinib in combination with capecitabine plus oxaliplatin in human epidermal growth factor receptor 2–positive advanced or metastatic gastric, esophageal, or gastroesophageal adenocarcinoma: TRIO-013/LOGiC—a randomized phase III trial. J Clin Oncol. (2016) 34:443–51. doi: 10.1200/jco.2015.62.6598. PMID: 26628478

[19] Xu R-H Gomes DBD Chen J-S Lordick F Paulson AS Rha SY . ARTEMIDE-Gastric01: A phase 3 randomized study of rilvegostomig withfluoropyrimidine and trastuzumab deruxtecan (T-DXd) as first-line (1L) treatment for locallyadvanced or metastatic HER2-positive gastric or gastroesophageal junctioncancer (GC/GEJC). Am Soc Clin Oncol. (2025)43(16_suppl):TPS4204. doi: 10.1200/JCO.2025.43.16_suppl.TPS4204

[20] Kim DW Ponto L Pereira AAL Kim RD Kracht C Lauwers G . A phase II study of neratinib (Nera) in combination withchemotherapy/trastuzumab/pembrolizumab (C/T/P) in HER2 overexpressing gastroesophageal cancers(GECs). Am Soc Clin Oncol. (2025)43(4_suppl):TPS514. doi: 10.1200/JCO.2025.43.4_suppl.TPS514

[21] Stein A Paschold L Tintelnot J Goekkurt E Henkes S-S Simnica D . Efficacy of ipilimumab vs FOLFOX in combination with nivolumab and trastuzumab in patients with previously untreated ERBB2-positive esophagogastric adenocarcinoma: the AIO INTEGA randomized clinical trial. JAMA Oncol. (2022) 8:1150–8. doi: 10.1001/jamaoncol.2022.2228. PMID: 35737383 PMC9227706

[22] Catenacci D Kang Y-K Yoon H Shim B Kim S Oh D-Y . Margetuximab with retifanlimab as first-line therapy in HER2+/PD-L1+ unresectable or metastatic gastroesophageal adenocarcinoma: MAHOGANY cohort A. ESMO Open. (2022) 7:100563. doi: 10.1016/j.esmoop.2022.100563. PMID: 36029651 PMC9588876

[23] Lee K-W Bai L-Y Jung M Ying J Im Y-H Oh D-Y . 1518P Zanidatamab (zani) plus chemotherapy (chemo) and tislelizumab (tis) as first-line (1l) therapy for patients (pts) with advanced HER2-positive (+) gastric/gastroesophageal junction adenocarcinoma (GC/GEJC): Updated results from a phase Ib/II study. Ann Oncol. (2023) 34:S855–6. doi: 10.1016/j.annonc.2023.09.1431. PMID: 41878731

[24] Elimova E Ajani J Burris H Denlinger CS Iqbal S Kang Y-K . Zanidatamab plus chemotherapy as first-line treatment for patients with HER2-positive advanced gastro-oesophageal adenocarcinoma: primary results of a multicentre, single-arm, phase 2 study. Lancet Oncol. (2025) 26(7):847–859. doi: 10.1016/s1470-2045(25)00287-6. PMID: 40473445 PMC13293637

[25] Tabernero J Shen L Elimova E Ku G Liu T Shitara K . HERIZON-GEA-01: Zanidatamab+ chemo ± tislelizumab for 1L treatment of HER2-positive gastroesophageal adenocarcinoma. Future Oncol. (2022) 18:3255–66. doi: 10.2217/fon-2022-0595. PMID: 36000541 PMC13238466

[26] Shen L Gong J Niu Z Zhao R Chen L Liu L . 1210P The preliminary efficacy and safety of KN026 combined with KN046 treatment in HER2-positive locally advanced unresectable or metastatic gastric/gastroesophageal junction cancer without prior systemic treatment in a phase II study. Ann Oncol. (2022) 33:S1102. doi: 10.1016/j.annonc.2022.07.1328. PMID: 41878731

[27] Liu D Gong J Li J Qi C Niu Z Liu B . Efficacy and safety of KN026, a bispecific anti-HER2 antibody, in combination with KN046, an anti-CTLA4/PD-L1 antibody, in patients with advanced HER2-positive nonbreast cancer: a combined analysis of a phase Ib and a phase II study. Signal Transduct Target Ther. (2025) 10:104. doi: 10.1038/s41392-025-02195-x. PMID: 40108113 PMC11923254

[28] Kong Y Dong Q Jin P Li M-Y Ma L Yi Q-J . Inetetamab combined with S-1 and oxaliplatin as first-line treatment for human epidermal growth factor receptor 2-positive gastric cancer. World J Gastroenterol. (2024) 30:4367. doi: 10.3748/wjg.v30.i40.4367. PMID: 39494102 PMC11525863

[29] Gong J Shen L Dong Z Liu D Xu J Yang J . 810 Preliminary safety, tolerability and efficacy results of KN026 in combination with KN046 in patients with HER2 aberrated solid tumors. BMJ Specialist J. (2020).

[30] Wei H Cai H Jin Y Wang P Zhang Q Lin Y . Structural basis of a novel heterodimeric Fc for bispecific antibody production. Oncotarget. (2017) 8:51037. doi: 10.18632/oncotarget.17558. PMID: 28881627 PMC5584228

[31] Xu J Ying J Liu R Wu J Ye F Xu N . KN026 (anti-HER2 bispecific antibody) in patients with previously treated, advanced HER2-expressing gastric or gastroesophageal junction cancer. Eur J Cancer. (2023) 178:1–12. doi: 10.1016/j.ejca.2022.10.004. PMID: 36370604

[32] Thuss-Patience PC Shah MA Ohtsu A Van Cutsem E Ajani JA Castro H . Trastuzumab emtansine versus taxane use for previously treated HER2-positive locally advanced or metastatic gastric or gastro-oesophageal junction adenocarcinoma (GATSBY): an international randomised, open-label, adaptive, phase 2/3 study. Lancet Oncol. (2017) 18:640–53. doi: 10.1016/s1470-2045(17)30111-0. PMID: 28343975

[33] Satoh T Xu R-H Chung HC Sun G-P Doi T Xu J-M . Lapatinib plus paclitaxel versus paclitaxel alone in the second-line treatment of HER2-amplified advanced gastric cancer in Asian populations: TyTAN—a randomized, phase III study. J Clin Oncol. (2014) 32:2039–49. doi: 10.1200/jco.2013.53.6136. PMID: 24868024

[34] Janjigian YY Ku GY Ilson DH Boyar MS Capanu M Chou JF . A phase II study of afatinib in patients (pts) with metastatic humanepidermal growth factor receptor (HER2)-positive trastuzumab refractory esophagogastric (EG)cancer. Am Soc Clin Oncol. (2015)74(19_Supplement):CT228-CT. doi: 10.1158/1538-7445.AM2014-CT228

[35] Kim CG Jung M Kim HS Lee C Jeung H-C Koo D-H . Trastuzumab combined with ramucirumab and paclitaxel in patients with previously treated human epidermal growth factor receptor 2–positive advanced gastric or gastroesophageal junction cancer. J Clin Oncol. (2023) 41:4394–405. doi: 10.1200/jco.22.02122. PMID: 37364218

[36] Makiyama A Sukawa Y Kashiwada T Kawada J Hosokawa A Horie Y . Randomized, phase II study of trastuzumab beyond progression in patients with HER2-positive advanced gastric or gastroesophageal junction cancer: WJOG7112G (T-ACT Study). J Clin Oncol. (2020) 38:1919–27. doi: 10.1200/jco.19.03077. PMID: 32208960

[37] Catenacci DV Kang Y-K Park H Uronis HE Lee K-W Ng MC . Margetuximab plus pembrolizumab in patients with previously treated, HER2-positive gastro-oesophageal adenocarcinoma (CP-MGAH22–05): A single-arm, phase 1b–2 trial. Lancet Oncol. (2020) 21:1066–76. doi: 10.1016/s1470-2045(20)30326-0. PMID: 32653053

[38] Xu J Liu R Ying J Wu J Ye F Xu N . A phase II study evaluating KN026 monotherapy in patients (pts) withpreviously treated, advanced HER2-expressing gastric or gastroesophageal junction cancers(GC/GEJC). Am Soc Clin Oncol. (2022)40(16_suppl):4040. doi: 10.1200/JCO.2022.40.16_suppl.4040

[39] Xu J Zhao J Chen Y Liu B Du Y Cheng Y . 1425P Efficacy and safety of KN026 in combination with chemotherapy in patients (pts) with unresectable or metastatic HER2 positive gastric or gastroesophageal cancers (GC/GEJC) after first-line treatment with a trastuzumab-containing regimen. Ann Oncol. (2024) 35:S889. doi: 10.1016/j.annonc.2024.08.1491. PMID: 41878731

[40] Xu J Zhao J Liu Y Chen Y Li S Cheng Y . LBA78 KN026 in combination with chemotherapy for previously treated HER2-positive gastric or gastroesophageal carcinomas (GC/GEJC): Interim analysis of KC-WISE. Ann Oncol. (2025) 36:S1736. doi: 10.1016/j.annonc.2025.09.093. PMID: 41878731

[41] Ku G Piha-Paul S Gupta M Oh D Kim Y Lee J . P-53 A phase 2, multi-center, open-label study of cinrebafusp alfa (PRS-343) in patients with HER2-high and HER2-low gastric or gastroesophageal junction (GEJ) adenocarcinoma. Ann Oncol. (2022) 33:S265. doi: 10.1016/j.annonc.2022.04.143. PMID: 41878731

[42] Shitara K Bang Y-J Iwasa S Sugimoto N Ryu M-H Sakai D . Trastuzumab deruxtecan in previously treated HER2-positive gastric cancer. N Engl J Med. (2020) 382:2419–30. doi: 10.1056/nejmoa2004413. PMID: 32469182

[43] Ku G Di Bartolomeo M Smyth E Chau I Park H Siena S . 1205MO Updated analysis of DESTINY-Gastric02: A phase II single-arm trial of trastuzumab deruxtecan (T-DXd) in western patients (Pts) with HER2-positive (HER2+) unresectable/metastatic gastric/gastroesophageal junction (GEJ) cancer who progressed on or after trastuzumab-containing regimen. Ann Oncol. (2022) 33:S1100. doi: 10.1016/j.annonc.2022.07.1323. PMID: 41878731

[44] Shitara K Gumus M Pietrantonio F Lonardi S De La Fouchardiere C Coutzac C . Trastuzumab deruxtecan (T-DXd) vs ramucirumab (RAM)+ paclitaxel(PTX) in second-line treatment of patients (pts) with human epidermal growth factor receptor2-positive (HER2+) unresectable/metastatic gastric cancer (GC) or gastroesophageal junctionadenocarcinoma (GEJA): Primary analysis of the randomized, phase 3 DESTINY-Gastric04study. Am Soc Clin Oncol. (2025)43(17_suppl):LBA4002-LBA. doi: 10.1200/JCO.2025.43.17_suppl.LBA4002

[45] Shen L Chen P Lu J Wan Y Zheng Y Ye F . 129MO Trastuzumab deruxtecan (T-DXd) in Chinese patients (pts) with previously treated HER2-positive (HER2+) advanced gastric or gastroesophageal junction adenocarcinoma (GEJA): DESTINY-Gastric06 (DG-06) final analysis. Ann Oncol. (2024) 35:S1454–5. doi: 10.1016/j.annonc.2024.10.155. PMID: 41878731

[46] Wang Y Gong J Wang A Wei J Peng Z Wang X . Disitamab vedotin (RC48) plus toripalimab for HER2-expressing advanced gastric or gastroesophageal junction and other solid tumours: a multicentre, open label, dose escalation and expansion phase 1 trial. EClinicalMedicine. (2024) 68:102415. doi: 10.1016/j.eclinm.2023.102415. PMID: 38235421 PMC10789637

[47] Peng Z Liu T Wei J Wang A He Y Yang L . Efficacy and safety of a novel anti‐HER2 therapeutic antibody RC48 in patients with HER2‐overexpressing, locally advanced or metastatic gastric or gastroesophageal junction cancer: a single‐arm phase II study. Cancer Commun. (2021) 41:1173–82. doi: 10.1002/cac2.12214. PMID: 34665942 PMC8626607

[48] Zhang Y Qiu M-Z Wang J-F Zhang Y-Q Shen A Yuan X-L . Phase 1 multicenter, dose-expansion study of ARX788 as monotherapyin HER2-positive advanced gastric and gastroesophageal junction adenocarcinoma.Cell Rep Med. (2022) 3(11):100814. doi: 10.1016/j.xcrm.2022.100814. PMID: 36384091 PMC9729820

[49] Strickler JH Nakamura Y Yoshino T Catenacci DV Janjigian YY Barzi A . MOUNTAINEER-02: Phase II/III study of tucatinib, trastuzumab,ramucirumab, and paclitaxel in previously treated HER2+ gastric or gastroesophageal junctionadenocarcinoma—Trial in Progress. Am Soc Clin Oncol.(2021) 40(4_suppl):TPS371-TPS. doi: 10.1200/JCO.2022.40.4_suppl.TPS371

[50] Shitara K Wainberg ZA Tabernero J Van Cutsem E Coutzac C De La Fouchardiere C . Final analysis of the randomized phase 2 part of the ASPEN-06 study:A phase 2/3 study of evorpacept (ALX148), a CD47 myeloid checkpoint inhibitor, in patients withHER2-overexpressing gastric/gastroesophageal cancer (GC). Am Soc ClinOncol. (2025) 43(4_suppl):332. doi: 10.1200/JCO.2025.43.4_suppl.332

[51] Yao H Yan M Tong Z Wu X Ryu M-H Park JJ . Safety, efficacy, and pharmacokinetics of SHR-A1811, a human epidermal growth factor receptor 2–directed antibody-drug conjugate, in human epidermal growth factor receptor 2–expressing or mutated advanced solid tumors: a global phase I trial. J Clin Oncol. (2024) 42:3453–65. doi: 10.1200/jco.23.02044. PMID: 38900984

[52] Jiang C Zhang L Xu X Qi M Zhang J He S . Engineering a smart agent for enhanced immunotherapy effect by simultaneously blocking PD‐L1 and CTLA‐4. Adv Sci. (2021) 8:2102500. doi: 10.1002/advs.202102500. PMID: 34473430 PMC8529437

[53] Catenacci DVT Lim KH Uronis HE Kang Y-K Ng MCH Gold PJ . Antitumor activity of margetuximab (M) plus pembrolizumab (P) in patients (pts) with advanced HER2+ (IHC3+) gastric carcinoma (GC). (2019) 37:65. doi: 10.1200/jco.2019.37.4_suppl.65

